# Targeted Radionuclide Therapy of Cancer and Infections

**DOI:** 10.3390/ijms24109081

**Published:** 2023-05-22

**Authors:** Bart C. H. van der Wal, Ekaterina Dadachova

**Affiliations:** 1 Department of Orthopedics, University Medical Center Utrecht, 3584 CX Utrecht, The Netherlands; b.c.h.vanderwal@umcutrecht.nl; 2 College of Pharmacy and Nutrition, University of Saskatchewan, Saskatoon, SK S7N 5A8, Canada

Targeted radionuclide therapy (TRT) has been burgeoning worldwide, with several radiopharmaceuticals for the treatment of metastatic cancers being approved for clinical use. There are many promising novel radiopharmaceuticals currently in preclinical development, and several are already being evaluated in Phase 0 or Phase 1 clinical trials. Many research groups are performing groundbreaking radiobiological and mechanistic studies in order to elucidate the mechanisms underpinning the efficacy of TRT, with the goal of making this modality more effective and safe. In addition, the recent COVID-19 pandemic has demonstrated the need for “out of the box” thinking when it comes to novel therapeutics for combating emerging infectious diseases. In this regard, applying the principles of TRT to the treatment of a whole plethora of infectious diseases seems to be an attractive approach.

This Special Issue, “Targeted Radionuclide Therapy of Cancer and Infections”, in the *International Journal of Molecular Sciences* includes six contributions—four original articles and two reviews—covering a very broad range of topics, from making radioastatinated precursors to the radiobiological mechanisms of TRT to evaluating radiopharmaceuticals for cancer and infection in animal models to clinical trials and approved radiopharmaceuticals for cancer therapy.

In the area of radiopharmaceutical chemistry, Li et al. [1] examined an alternative approach of making precursors for the labeling of molecules with astatine-211 (211At), as this alpha emitter has a lot of promise for TRT. Specifically, the authors tested the hypothesis that a specific type of astatinated aryl compound with highly oxidized At atoms could exhibit stability in vivo towards deastatination. To accomplish this, para-stannylbenzoic acid derivatives were synthesized and radiolabeled with 125I and 211At, followed by the oxidization of the [125I]iodo and [211At]astato- benzamidyl-dPEG4-acid methyl ester derivatives for subsequent biodistribution in mice. While the oxidized radioiodinated derivative did not deiodinate in vivo, the oxidized [211At]astatinated benzamide derivative was shown to be unstable in vivo. These results highlight many challenges in developing reliable chemistry for making stable radiopharmaceuticals for TRT, especially the ones that incorporate radionuclides with very complex chemistry, such as At.

Detailed knowledge of radiobiological mechanisms of action of TRT agents is very important for developing novel radiopharmaceuticals, as well as for designing more effective and safe treatment regimens. Though TRT has been in development for several decades, many radiobiological mechanisms are still extrapolated from external beam radiation therapy (EBRT) in spite of stark differences in doses, dose rates, and times of administration between these two radiation treatment modalities. Delbart et al. [2] described their in vitro experience in comparing several major important radiobiological outcomes, such as cell survival, cell cycle, cell death, oxidative stress, and DNA damage in six human cancer cell lines for a TRT agent and for EBRT. In addition, they investigated the radiosensitization effect of olaparib, an inhibitor of a poly (ADP-ribose) polymerase (PARP) in combination with TRT and EBRT. As a TRT agent, they utilized 177Lu-DOTATATE—a peptide that binds to somatostatin receptors in neuroendocrine tumors and has been approved for use in clinical practice. Their study demonstrated the significant similarity of radiobiological mechanisms through which both 177Lu-DOTATATE and EBRT killed cells, but the extent of this killing and its kinetics, as well as the extent of DNA damage, were different between TRT and EBRT. In addition, radiosensitization with olaparib was more pronounced in combination with EBRT than with TRT. These results convincingly prove the need for continuous radiobiological studies of TRT mechanisms, which will help in designing more effective combination treatments.

The paper by Sreekumar et al. [3] describes the development of TRT for prostate cancer in prostate cancer xenograft models. Interestingly, their target is similar to one of the targets in [2]—it is poly (ADP-ribose) polymerase-1 (PARP-1) that is overexpressed in prostate cancer. Since PARP-1 is located close to a cell’s DNA, researchers hypothesized that it would be a suitable target for a TRT agent carrying an Auger radiation-emitting radionuclide that could cause double-strand breaks in DNA in prostate cancer cells. For that purpose, they synthesized a radio-brominated Auger electron-emitting PARP-1 inhibitor ([77Br]Br-WC-DZ) and used it in vitro and in vivo to assess its efficacy as well as mechanisms of action. There was a strong correlation between the levels of PARP-1 expression and Gleason score in a prostate cancer tumor microarray, which is important for developing this agent into a therapeutic for advanced prostate cancer, especially in patients who are not the candidates for treatment with 177Lu-PMSA-617 (Pluvicto).

A review by Allen et al. [4] summarizes the recent developments in the area of radiopharmaceuticals targeting melanin, a pigment that gives melanoma cancer its name, in metastatic melanoma. The review covers two approaches to targeting melanin—with melanin-specific monoclonal antibodies (radioimmunotherapy (RIT)) and with melanin-binding radiolabeled small molecules. RIT with melanin-specific antibodies radiolabeled with an alpha emitter, 213Bi, or beta emitter, 177Lu, showed encouraging results in experimental melanoma when it was combined with immunotherapy utilizing anti-PD-1 antibodies. Radioiodinated benzamides are small molecules that can cross the cellular membranes and bind to intracellular melanin. In this way, they can be used for both nuclear imaging and TRT. Extensive preclinical work has identified a promising lead, [131I]ICF01012, which is currently in clinical trials in melanoma patients (NCT03784625). The continuation of both preclinical and clinical work on melanin-targeting TRT will, hopefully, lead to its development as a monotherapy or a combinatorial therapy in conjunction with standard-of-care drugs.

A review by van Nuland et al. [5] analyzes the radiopharmaceutical dosing and pharmacokinetics of already-approved TRT drugs, such as 186Re-hydroxyethylidene diphosphonate (HEDP), 131I sodium iodide, 131I-metaiodobenzylguanidine (MIBG), 177Lu-DOTATATE, and 177Lu-PSMA-617, in obese patients. This is an important topic as the number of obese patients is increasing each year in the Western population and worldwide, and the absence of dosing guidelines in such patients could lead to under- or overdosing, resulting in less effective treatment or toxicity, respectively. A data analysis revealed that fixed dosing in obese patients may be appropriate for these approved TRT drugs. The authors emphasized the need for more research in order to create the guidelines for TRT administration in obese patient populations.

The paper by van Dijk et al. [6] is different from other papers in this Special Issue, as it describes developing the radioimmunoimaging of *Staphylococcus aureus* infection in a mouse implant infection model. There is a clinical need to treat prosthetic joint infections (PJI) in patients as implant infections caused by *S. aureus* biofilms are difficult to treat, thus complicating treatment with repeat surgery and antibiotics. To accomplish this, researchers radiolabeled with 111In a monoclonal antibody, 4497, which binds to the teichoic acid in the cell wall of *S. aureus*, and performed the microSPECT/CT imaging of mice implanted with infected and sterile implants. They found that, at all time points, the 111In-4497 antibody accumulated significantly higher in infected than in sterile implants, thus confirming the specificity of targeting infection with this antibody. They concluded that this antibody might serve in the delivery of not only imaging, but also therapeutic radionuclides to sites of infection.

In conclusion, this Special Issue, “Targeted Radionuclide Therapy of Cancer and Infections”, covers different aspects of developing and using TRT in preclinical and clinical settings, and, hopefully, will contribute to attracting interested readers to this topic and to enhancing the growth of TRT field.
